# Novel candidate metastasis‐associated genes for synovial sarcoma

**DOI:** 10.1111/jcmm.18541

**Published:** 2024-07-24

**Authors:** Zhiqing Zhao, Jianfang Niu, Jichuan Wang, Ranxin Zhang, Haijie Liang, Yingteng Ma, Alexander Ferrena, Wei Wang, Rui Yang, David S. Geller, Wei Guo, Tingting Ren, Bang H. Hoang, Xiaodong Tang, Taiqiang Yan

**Affiliations:** ^1^ Department of Orthopedics Peking University First Hospital Beijing China; ^2^ Musculoskeletal Tumor Center Peking University People's Hospital Beijing China; ^3^ Beijing Key Laboratory of Musculoskeletal Tumor Beijing China; ^4^ Department of Orthopedic Surgery, Montefiore Medical Center Albert Einstein College of Medicine Bronx New York USA; ^5^ Department of Pathology Peking University People's Hospital Beijing China; ^6^ Department of Genetics, Institute for Clinical and Translational Research Albert Einstein College of Medicine Bronx New York USA

**Keywords:** *EXO1*, metastasis, *NCAPG*, *POLQ*, synovial sarcoma, *UHRF1*

## Abstract

Synovial sarcoma (SS) is an aggressive soft tissue sarcoma with poor prognosis due to late recurrence and metastasis. Metastasis is an important prognostic factor of SS. This study aimed to identify the core genes and mechanisms associated with SS metastasis. Microarray data for GSE40021 and GSE40018 were obtained from the Gene Expression Omnibus database. 186 differentially expressed genes (DEGs) were identified. The biological functions and signalling pathways closely associated with SS metastasis included extracellular matrix (ECM) organization and ECM‐receptor interaction. Gene set enrichment analysis showed that the terms cell cycle, DNA replication, homologous recombination and mismatch repair were significantly enriched in the metastasis group. Weighted gene co‐expression network analysis identified the most relevant module and 133 hub genes, and 31 crossover genes were identified by combining DEGs. Subsequently, four characteristic genes, *EXO1*, *NCAPG*, *POLQ* and *UHRF1*, were identified as potential biomarkers associated with SS metastasis using the least absolute shrinkage and selection operator algorithm and validation dataset verification analysis. Immunohistochemistry results from our cohort of 49 patients revealed visible differences in the expression of characteristic genes between the non‐metastatic and metastatic groups. Survival analysis indicated that high expression of characteristic genes predicted poor prognosis. Our data revealed that primary SS samples from patients who developed metastasis showed activated homologous recombination and mismatch repair compared to samples from patients without metastasis. Furthermore, *EXO1*, *NCAPG*, *POLQ* and *UHRF1* were identified as potential candidate metastasis‐associated genes. This study provides further research insights and helps explore the mechanisms of SS metastasis.

## 1 INTRODUCTION

Synovial sarcoma (SS) is an aggressive neoplasm that accounts for 5%–10% of soft tissue sarcomas in adolescents and young adults.^1^ SS is characterized by a specific *t*(X;18) (p11.2; q11.2) chromosomal translocation that generates the SYT‐SSX (also known as SS18‐SSX) fusion, an oncogene that can be detected in more than 95% of cases of SS.^2^ Although SS can occur anywhere in the soft tissues of the body, 70% develop in the extremities.^3^ SS displays a high degree of aggressiveness and tendency to metastasize. Late metastasis occurs in 50%–70% of patients, and <10% of patients have detectable metastases at the time of diagnosis.^4^, ^5^, ^6^ The most frequent metastatic sites are the lungs (80%), followed by the bone (9.9%) and liver (4.5%).^7^ The mainstay of curative therapy is surgical resection of the localized disease, with or without radiotherapy.^8^ Although the 5‐year survival rate is 59.4%, it decreases to 50.8% at 10 years and 42.8% at 20 years, owing to the high incidence of late metastases.^9^ Patients with metastases at the time of diagnosis had a significantly worse prognosis, with a 3‐year survival rate of 27.2%.^10^ Therefore, metastasis is an important prognostic factor of SS that deserves more attention and research.

Several molecular alterations associated with SS have been identified and used as potential targets for therapy, including SS18‐SSX,^11^ IGF‐1R,^12^ BCL2,^13^ TLE1^14^ and Wnt/β catenin pathway components (LEF1, TCF7, ZIC2, WNT5A, AXIN2 and FZD10).^12^ TLE1 is one of the most commonly overexpressed genes in SS, and is therefore used as a marker to distinguish SS from other soft tissue sarcomas.^14^, ^15^ BCL2 is consistently highly expressed in SS and is thought to induce an antiapoptotic phenotype.^13^ Receptor tyrosine kinases (RTKs), such as vascular endothelial growth factor receptors (VEGFRs) and platelet‐derived growth factor receptors (PDGFRs), are also involved in SS progression.^16^, ^17^ Clinical trials have been carried out to inhibit these targets, such as using the tyrosine kinase receptor inhibitor pazopanib^18^, ^19^ and the IGF‐1R antibody cixutumumab.^20^ Although VEGFR, PDGFR and IGF‐1R have shown promise in preclinical studies, the effect of inhibition of these targets on improving clinical outcomes remains limited.^20^, ^21^ Metastasis of SS is a major factor responsible for poor prognosis. Therefore, elucidating the mechanisms underlying the development and metastasis of SS and discovering new therapeutic targets related to metastasis are important to improve the prognosis of patients with SS.

In our study, we carried out bioinformatic analyses leveraging publicly available gene expression datasets to investigate the mechanisms underlying the metastasis of SS and identify candidate genes associated with metastasis. Our data revealed that primary samples that developed metastasis showed activated homologous recombination and mismatch repair compared to samples without metastasis. We identified four characteristic genes related to metastasis, which provides new research directions for inhibiting SS metastasis.

## 2 MATERIALS AND METHODS

### 2.1 Data acquisition and screening for differentially expressed genes (DEGs)

Microarray expression data of SS were downloaded from the Gene Expression Omnibus (GEO) database. Dataset GSE40021, which was used as the training set, included 30 primary samples from patients without metastasis and 28 with metastasis. In the validation dataset, GSE40018, there were 17 primary samples from patients without metastasis and 17 with metastasis. The platform probe annotation files were also downloaded, and the probes were converted into the corresponding gene symbols using *Perl* language. The R package *limma* was used to screen DEGs between primary SS samples from patients with and without metastasis, with |log2 fold change (FC)| > 1.5 and adjusted *p*‐value <0.05.

### 2.2 Functional enrichment analysis

Gene Ontology (GO) functional enrichment analyses, including biological process (BP), cellular component (CC), molecular function (MF) and Kyoto Encyclopedia of Genes and Genomes (KEGG) pathway enrichment analyses of the DEGs, were performed using the R package *clusterProfiler*. GO and KEGG terms with *p* < 0.05 were considered significant. Gene Set Enrichment Analysis (GSEA) was performed on the OECloud platform (https://cloud.oebiotech.cn/task/). The absolute value of normalized enrichment score (NES) > 1, *p* < 0.05, and false discovery rate (FDR) <0.25 were considered statistically significant for GSEA.

### 2.3 WGCNA analysis

A weighted gene co‐expression network analysis (WGCNA) was performed to identify modules highly correlated with the metastatic feature of SS in the GSE40021 dataset. We applied the WGCNA R package to cluster all genes based on co‐expression patterns to construct the gene co‐expression network, which was transformed into an adjacency matrix and then into a topological overlap matrix (TOM). Based on the TOM‐based dissimilarity measure, genes with similar expression profiles were clustered into the same module using average‐linkage hierarchical clustering. Here, the soft‐thresholding power was set to 5, the minimal module size was 60, and the cut height was 0.25. Finally, we tested the correlation between the modules and the metastatic features using Pearson's correlation coefficient, at a significance level of 0.05.

### 2.4 Characteristic gene identification

After identifying the highly correlated module, genes with gene significance >0.5 and module membership >0.8 were taken as hub genes in WGCNA. Candidate genes were identified by intersecting DEGs and WGCNA hub genes using the *venn* package. Finally, the least absolute shrinkage and selection operator (LASSO) logistic regression analysis was performed using the *glmnet* package to screen the characteristic genes from the candidate genes.

### 2.5 Verification of characteristic genes

The *ggpubr* package was used to visualize the differential expression of characteristic genes between the non‐metastatic and metastatic groups. Receiver operating characteristic (ROC) curve analysis was performed using the *pROC* package to determine the area under the curve (AUC) of characteristic genes.

### 2.6 Tissue samples

SS tissue samples for immunohistochemistry (IHC) were obtained from 49 patients at the Musculoskeletal Tumour Center, Peking University People's Hospital. The study protocol was approved by the Ethics Committee of Peking University People's Hospital, and informed consent was obtained from all participants. The SS tissue microarray was purchased from Bioaitech Co., Ltd (K024Sf01). Twenty‐three samples (9 T1 and 14 T2) were used in the study, excluding one without data about invasiveness.

### 2.7 Immunohistochemistry

Immunohistochemistry was performed as previously described.^22^ Briefly, formalin‐fixed, paraffin‐embedded tumour tissue sections were stained with primary antibodies against EXO1 (PA5‐86470; Invitrogen), NCAPG (PA5‐58857; Invitrogen), POLQ (ab111218; Abcam) or UHRF1 (ab213223; Abcam). The expression of these markers in the samples was evaluated based on the staining intensity and percentage of positively stained cells. The intensity score was defined as follows: 0, negative staining; 1, weak staining; 2, moderate staining; 3, strong staining. The percentage of positively stained cells was scored as: 0, <5%; 1, 5%–25%; 2, 26%–50%; 3, 51%–75%; and 4, >75%. The IHC score (H‐score) was calculated by multiplying the intensity and percentage scores of the positive cells, and the final H‐score of each sample was calculated by averaging the scores of five randomly selected areas.

### 2.8 Survival analysis

Survival analyses were performed using the Gene Expression Profiling Interactive Analysis (GEPIA) Database (http://gepia.cancer‐pku.cn/). Based on the median gene expression level, the samples were divided into high‐ and low‐expression groups. The overall survival and disease‐free survival were estimated using the log‐rank test.

### 2.9 Statistical analysis

Statistical analyses were performed using Prism 8. Statistically significant differences between groups were determined using the Student's *t* test. Data are presented as mean ± standard error of the mean (SEM). Differences were considered statistically significant at *p* < 0.05, and significance levels were assigned as follows: *p < 0.05, **p < 0.01, ***p < 0.001, ns, not significant.

## 3 RESULTS

### 3.1 Identification of DEGs and functional enrichment analysis

**FIGURE 1:**
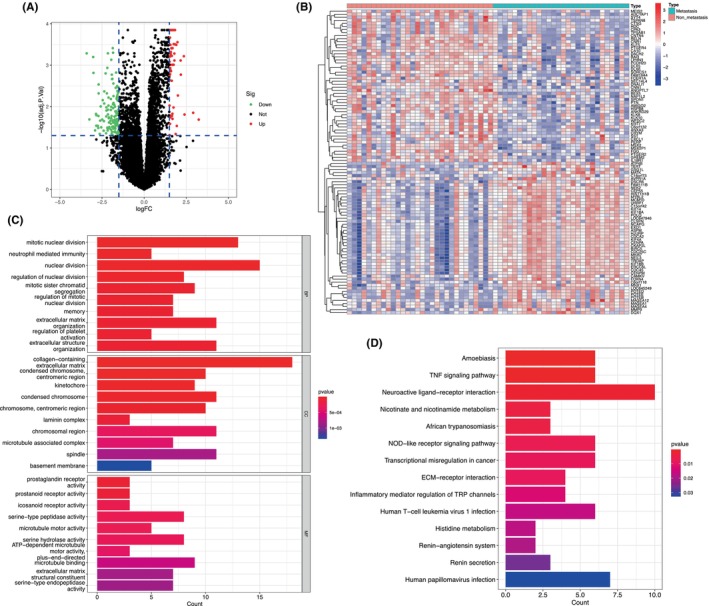
Identification and functional enrichment analyses of DEGs. (A) Volcano plot of DEGs. The red and green dots represent the significantly upregulated and downregulated genes, respectively. The black dots represent genes with no significant change. (B) Heatmap of the top 50 significantly upregulated and downregulated genes between the non‐metastatic and metastatic groups. (C) GO enrichment analysis of the DEGs involved in biological processes (BP), cellular components (CC), and molecular functions (MF). (D) KEGG pathway analysis.

Dataset GSE40021 was used to screen DEGs in the non‐metastatic and metastatic groups. Based on the cutoff criteria of |log_2_ FC| > 1.5 and an adjusted *p*‐value <0.05, 186 DEGs were identified, including 54 upregulated and 132 downregulated genes (Figure 1A and Table S1). The heatmap shows the top 50 upregulated and downregulated DEGs (Figure 1B). Functional analyses of the DEGs were performed using GO and KEGG functional enrichment analyses, and the results indicated that these genes were enriched in functions associated with extracellular matrix (ECM) organization and ECM‐receptor interaction (Figure 1C,D).

### 3.2 GSEA analysis

**FIGURE 2:**
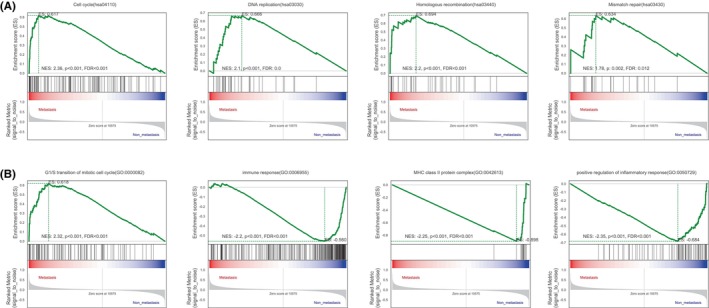
GSEA analysis. (A**)** Enrichment of pathways based on the KEGG database. **(**B**)** Enrichment of pathways based on the GO database.

We conducted GSEA analysis using the KEGG database to identify several signalling pathways altered between the non‐metastatic and metastatic groups. The data revealed that the cell cycle, DNA replication, homologous recombination and mismatch repair were significantly enriched pathways in the SS metastatic group (Figure 2A). GSEA based on the GO database also showed enrichment of the gene set involved in the G1/S transition of the mitotic cell cycle in the metastatic group. In addition, GSEA revealed several gene sets associated with immune response, MHC class II protein complex and positive regulation of the inflammatory response, which were significantly enriched in the non‐metastatic group (Figure 2B).

### 3.3 Identification of characteristic genes

**FIGURE 3:**
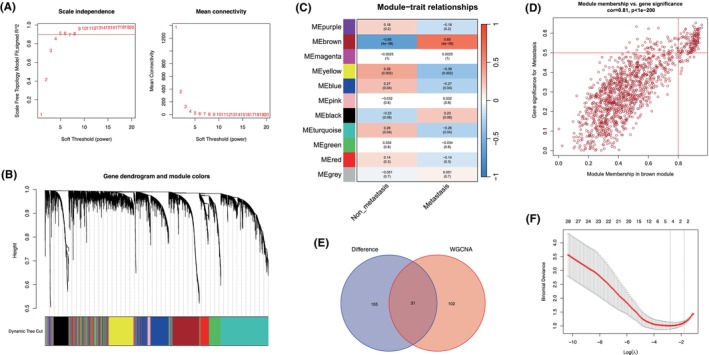
Identification of modules and characteristic genes associated with SS metastasis. (A) Identification of soft‐thresholding power in WGCNA analysis. (B) Cluster dendrogram of the identified co‐expression modules. (C) Heatmap of the correlation between identified modules and clinical traits. (D) Hub genes identified by module membership and gene significance in the brown module. (E) Venn diagram of co‐expressed genes between DEGs and hub genes. (F) Partial likelihood deviance for the LASSO regression.

To explore the functional modules related to SS metastatic potential, a weighted gene co‐expression network was constructed from the GSE40021 dataset, which contained 30 primary samples from patients without metastasis and 28 with metastasis. The samples were clustered using the average‐linkage hierarchical clustering method. We then identified the soft‐thresholding power β of five to construct a scale‐free network (Figure 3A). As a result, 11 co‐expression modules were identified by gathering similarly expressed genes (Figure 3B). To evaluate the association between each module and the metastatic potential, a heatmap of the module‐trait relationship was plotted. The module eigengene (ME) of the brown module was found to have the strongest correlation with metastatic traits (Figure 3C); therefore, the brown module was selected for subsequent analysis. Based on module membership >0.8 and gene significance >0.5, we screened 133 hub genes from the brown module (Figure 3D and Table S2). We then constructed an intersection of the DEGs and hub genes and identified 31 candidate genes (Figure 3E and Table S3). Using LASSO logistic regression analysis, we identified four characteristic genes, namely *EXO1* (exonuclease 1), *NCAPG* (non‐SMC condensin I complex subunit G), *POLQ* (DNA polymerase theta) and *UHRF1* (ubiquitin‐like with plant homeodomain and ring finger domains 1) (Figure 3F).

### 3.4 Validation of characteristic genes

**FIGURE 4:**
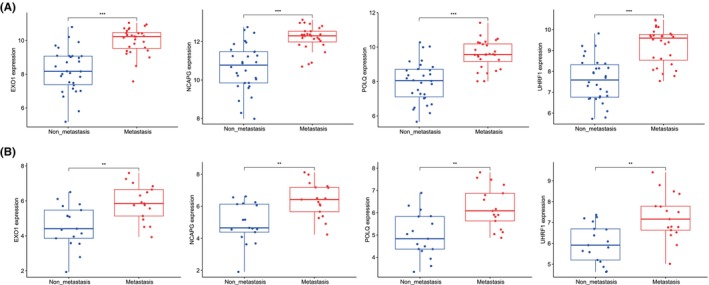
Comparison of the expression levels of characteristic genes between the non‐metastatic and metastatic groups. Expression of characteristic genes in the training dataset GSE40021 (A) and validation dataset GSE40018 (B). **p < 0.01, ***p < 0.001.

**FIGURE 5:**
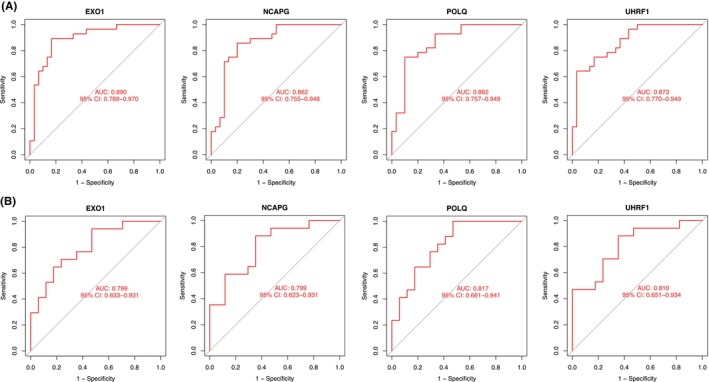
ROC curves for the characteristic genes. ROC curves for the four characteristic genes in the training dataset GSE40021 (A) and validation dataset GSE40018 (B).

We subsequently analysed the expression of the four characteristic genes in the non‐metastatic and metastatic groups in the training dataset GSE40021. The expression of these four genes was higher in the metastatic group than in the non‐metastatic group (Figure 4A). The same conclusion was drawn for the validation dataset GSE40018 (Figure 4B). To investigate the diagnostic value of the four characteristic genes, we performed ROC analysis. The results revealed that the AUC of each of these four genes in the training dataset GSE40021 was >0.8, suggesting high diagnostic accuracy (Figure 5A). AUC values >0.7 in the validation dataset GSE40018 also confirmed the diagnostic potency of the characteristic genes (Figure 5B).

### 3.5 Expression of characteristic genes and prognostic analysis

**FIGURE 6:**
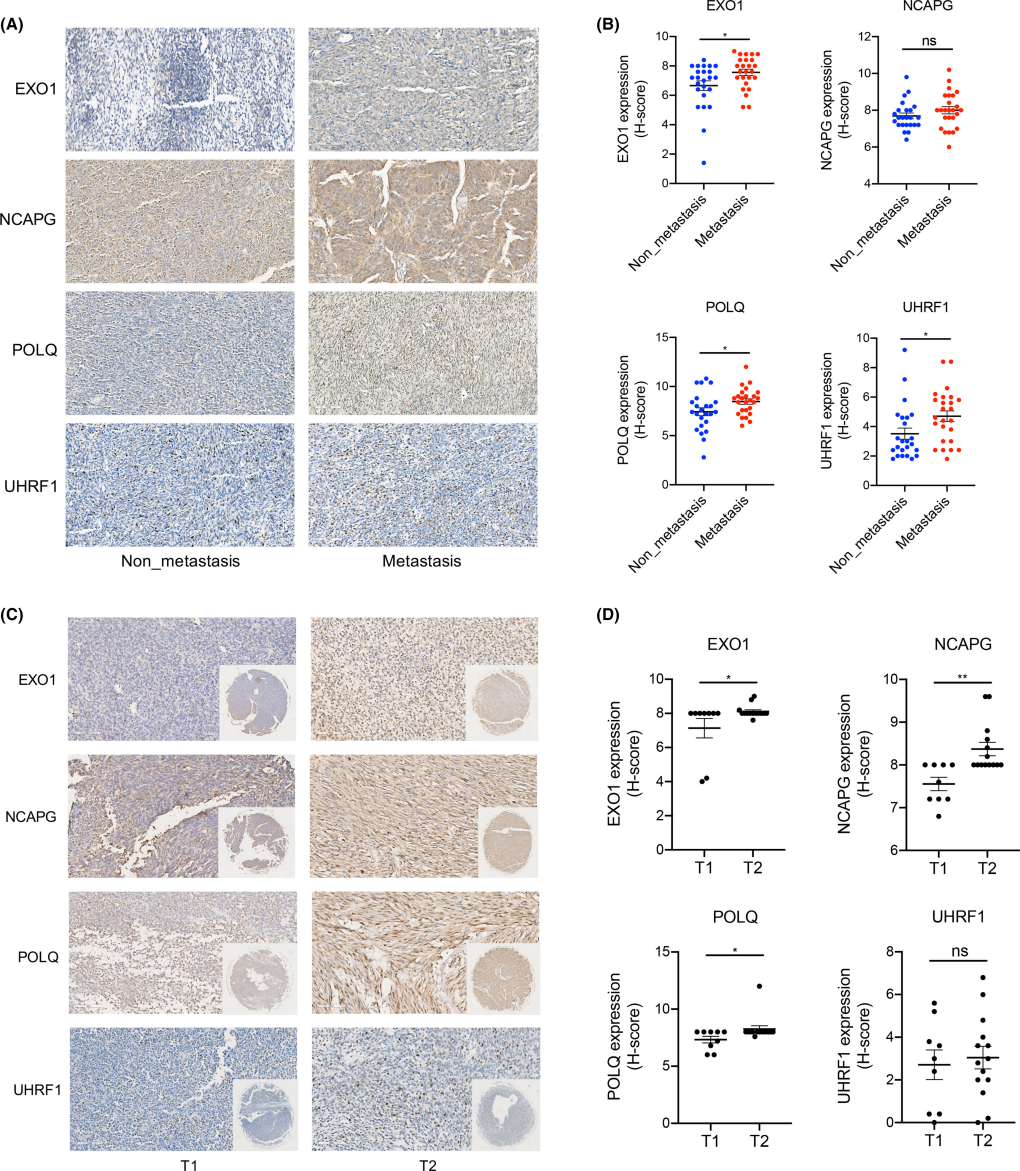
Expression of characteristic genes in SS tissue. (A) Immunohistochemical staining of EXO1, NCAPG, POLQ, and UHRF1 in primary SS tissues with (*n* = 25) and without (*n* = 24) metastasis. (B) Quantitative comparison of the immunohistochemical histoscores of EXO1, NCAPG, POLQ, and UHRF1 between the non‐metastatic and metastatic groups. (C) Immunohistochemical staining of EXO1, NCAPG, POLQ, and UHRF1 in non‐invasive (T1, *n* = 9) and invasive (T2, *n* = 14) tissues. (D) Quantitative comparison of the immunohistochemical histoscores of EXO1, NCAPG, POLQ, and UHRF1 between non‐invasive and invasive lesions. *p < 0.05, **p < 0.01, ns indicates not significant.

**FIGURE 7:**
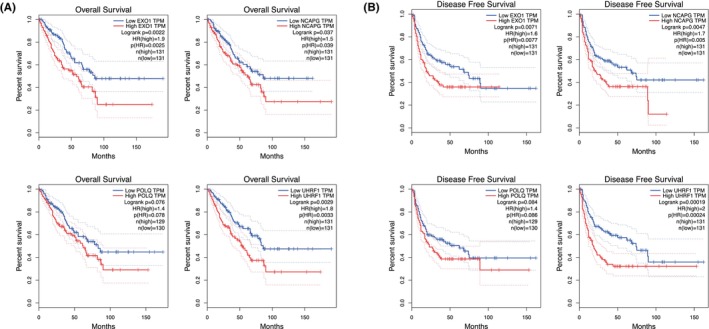
Survival analysis of the characteristic genes. Association between *EXO1*, *NCAPG*, *POLQ*, and *UHRF1* expression and overall survival (A) and disease‐free survival (B) using the log‐rank test.

To further validate the selected targets, we investigated protein expression using immunohistochemistry in a cohort of 49 patients with SS. Similar to the mRNA microarray results, the expression levels of EXO1, POLQ, and UHRF1 were significantly higher in the metastatic group than in the non‐metastatic group. The mean NCAPG expression level in the metastatic group was higher than that in the non‐metastatic group, although the difference was not statistically significant (Figure 6A,B). Moreover, we evaluated the relationship between the expression of characteristic genes and tumour invasiveness using a tissue microarray. The results showed that EXO1, NCAPG and POLQ were highly expressed in invasive lesions (T2). The expression of UHRF1 showed significant heterogeneity, and the mean level of UHRF1 expression was slightly higher in invasive lesions (T2) than in non‐invasive lesions (T1) (Figure 6C,D). To further determine the prognostic value of these four genes, we used the GEPIA database to investigate the relationship between gene expression and survival outcomes in patients with sarcoma. The results showed that patients with higher expression levels of *EXO1*, *NCAPG*, *POLQ* and *UHRF1* not only had a shorter overall survival but also a shorter disease‐free survival (Figure 7A,B). Taken together, these results indicate that these four genes may be associated with tumour invasiveness, metastasis and may affect patient survival.

## 4 DISCUSSION

To identify the characteristic genes associated with SS metastasis, we performed bioinformatic analyses of SS samples with or without metastasis based on transcriptome data. GSEA analysis with the functional annotation of genes and WGCNA with the selection of characteristic modules and hub genes were used to explore the mechanisms of metastasis in SS and the characteristic genes associated with metastasis.

We identified 54 upregulated and 132 downregulated genes between the primary samples from patients with metastasis and without metastasis. Functional analyses of the DEGs indicated that ECM organization and the interaction between the ECM and receptor play important roles in SS metastasis. GSEA revealed that genes were enriched for functions in homologous recombination and mismatch repair in the primary samples from patients who developed metastasis. Homologous recombination is a DNA repair mechanism responsible for the repair of double‐stranded breaks and the maintenance of genome integrity. A previous study showed that the induction or overexpression of the homologous recombination pathway leads to an increase in the number of new mutations and gene rearrangements, which contribute to the development of invasive, metastatic or drug‐resistant phenotypes.^23^ In our study, homologous recombination was enriched in the primary samples from patients with metastasis, suggesting that activation of homologous recombination may be one of the mechanisms promoting SS metastasis. Mismatch repair prevents the accumulation of mutations by repairing erroneous insertions, deletions and mismatch substitutions during DNA replication and transcription.^24^ Mutation or silencing of mismatch repair genes is associated with the accumulation of multiple mutations and microsatellite instability.^25^ One study defined the mismatch repair status of gastric cancer samples by detecting the expression of four mismatch repair proteins (MLH1, PMS2, MSH2 and MSH6) using immunohistochemistry. The absence of any of the four mismatch repair proteins was defined as deficient mismatch repair (dMMR), and further revealed that the dMMR status was significantly correlated with lower lymph node metastasis and good prognosis. The study indicated that increased tumour antigens and a large number of lymphocytes infiltrating dMMR tumours may be responsible for the better outcome.^26^ Yoon et al. also concluded that patients with sporadic colorectal cancer with dMMR had infrequent lymph node metastasis.^27^ In our study, samples from patients with metastasis exhibited a mismatch repair‐proficient status, indicating that the expression of mismatch repair proteins may be another mechanism promoting SS metastasis. However, further studies are required to confirm these conclusions.

In the present study, we screened four genes closely related to SS metastasis. EXO1, a member of the RAD2/XPG nuclease family, plays an important role in DNA damage repair, replication, and homologous recombination.^28^ Because EXO1 functions in a variety of DNA repair pathways, the relationship between this gene and cancer has previously been investigated. A previous study reported that overexpression of *EXO1* protects ovarian cancer cells from cisplatin‐mediated apoptosis, and inhibition of *EXO1* enhances chemotherapy cytotoxicity against ovarian cancer.^29^ In addition, *EXO1* is overexpressed in astrocytoma and is associated with an unfavourable prognosis.^30^ *EXO1* is also thought to play a role in the progression of hepatocellular carcinoma, and its high expression levels have been found to be positively related to tumour migration, invasion, and lymph node metastasis.^31^, ^32^ The expression level of EXO1 in our study was also significantly upregulated in primary SS samples from patients with metastasis compared to primary tumour samples from patients without metastasis. The results from the tissue microarray also indicated significantly higher EXO1 expression in invasive lesions. Survival analysis based on the GEPIA database showed that patients with high *EXO1* expression had a poor prognosis. Our data indicate that EXO1 is an important factor related to SS metastasis, and EXO1 may be a strong prognostic biomarker for SS.

NCAPG is a subunit of the condensin I complex that is responsible for the condensation and stabilization of chromosomes during mitosis and meiosis.^33^ The oncogenic role of *NCAPG* has been identified in several tumours, including prostate cancer, breast cancer, gliomas, gastric cancer and hepatocellular carcinoma.^34^, ^35^, ^36^, ^37^, ^38^ Moreover, the knockdown of *NCAPG* inhibits the proliferation, migration, and invasion of non‐small cell lung cancer (NSCLC) cells, and *NCAPG* overexpression is negatively associated with the survival of patients with NSCLC.^39^ Consistent with these studies, our microarray data demonstrated a significant increase in *NCAPG* expression in primary SS samples from patients with metastasis. At the protein level of NCAPG, as measured by immunohistochemistry, we observed that the mean expression of NCAPG was higher in the metastatic group than in the non‐metastatic group, but this difference was not statistically significant. However, the expression of NCAPG in invasive lesions was significantly higher than that in non‐invasive lesions. It is evident that NCAPG is involved in the progression of multiple tumours, and our study demonstrated that *NCAPG* expression was significantly correlated with overall survival and disease‐free survival; however, its role in the metastasis of SS requires further research.

POLQ is reported to be involved in the microhomology‐mediated end‐joining repair of double‐stranded DNA breaks.^40^ Several studies have indicated that *POLQ* is highly expressed in human cancers,^41^, ^42^ and its overexpression is linked to poor survival outcomes in breast cancer.^43^ Currently, there are few reports regarding the role of POLQ in cancer. In our study, we used publicly available data to clarify the differential expression of *POLQ* between the metastatic and non‐metastatic groups and further confirmed that POLQ is more highly expressed in primary SS samples from patients with metastasis than in the non‐metastatic group in our cohort. Further study also demonstrated that high expression of POLQ was associated with tumour invasiveness. Prognostic analysis revealed that high *POLQ* expression predicted poor survival, although the difference was not statistically significant. In conclusion, POLQ may play an important role in SS metastasis; however, further studies with more targeted experiments are required.

UHRF1 functions in DNA methylation and histone H3 ubiquitination.^44^, ^45^ Several studies have defined *UHRF1* as an oncogene in various tumours, including breast cancer, cervical squamous cell carcinoma, prostate cancer, and osteosarcoma. UHRF1 promotes tumour progression by regulating proliferation, apoptosis, migration, invasion, and metastasis.^46^, ^47^, ^48^, ^49^ In this study, we identified the differences in *UHRF1* mRNA and protein levels between primary SS samples from patients with and without metastasis. Immunohistochemical results from the tissue microarray showed a slightly higher expression of UHRF1 in invasive versus non‐invasive lesions. Moreover, our data revealed that high *UHRF1* expression was associated with poor overall survival and disease‐free survival. Therefore, UHRF1 may be served as a potential biomarker for predicting SS metastasis and patient prognosis.

## 5 CONCLUSIONS

Using microarray data from the GEO database and integrated WGCNA analysis, we investigated the mechanisms of SS metastasis and identified four metastasis‐associated genes. The terms homologous recombination and mismatch repair were significantly enriched in the group with metastasis, suggesting that these functions may play a role in SS metastasis. In addition, we identified *EXO1*, *NCAPG*, *POLQ* and *UHRF1* as novel candidate genes for SS metastasis and confirmed their high expression in invasive lesions and primary samples from patients with metastasis. Further research is necessary to elucidate the precise roles of these genes in SS metastasis and the underlying mechanisms. Taken together, our study explores the mechanisms associated with SS metastasis and proposes novel potential genes that are related to SS metastasis.

## AUTHOR CONTRIBUTIONS

**Zhiqing Zhao:** Data curation (equal); formal analysis (equal); investigation (equal); methodology (equal); writing – original draft (lead). **Jianfang Niu:** Data curation (equal); formal analysis (equal); investigation (equal); methodology (equal); writing – original draft (equal). **Jichuan Wang:** Data curation (equal); formal analysis (equal); funding acquisition (equal); investigation (equal); methodology (equal); resources (equal); writing – original draft (supporting). **Ranxin Zhang:** Investigation (equal); methodology (equal); resources (equal); software (lead). **Haijie Liang:** Formal analysis (equal); investigation (equal); software (equal); validation (lead). **Yingteng Ma:** Investigation (equal); methodology (equal); supervision (equal); validation (equal). **Alexander Ferrena:** Methodology (equal); resources (equal); software (equal); visualization (equal). **Wei Wang:** Investigation (equal); software (equal); validation (equal). **Rui Yang:** Formal analysis (equal); methodology (equal); software (equal); visualization (equal). **David S. Geller:** Methodology (equal); software (equal); visualization (equal). **Wei Guo:** Methodology (equal); supervision (equal); visualization (equal). **Tingting Ren:** Methodology (equal); validation (equal); visualization (equal). **Bang H. Hoang:** Funding acquisition (equal); methodology (equal); software (equal); visualization (equal). **Xiaodong Tang:** Conceptualization (equal); project administration (equal); supervision (equal); writing – review and editing (equal). **Taiqiang Yan:** Conceptualization (equal); funding acquisition (equal); project administration (lead); supervision (lead); writing – review and editing (lead).

## CONFLICT OF INTEREST STATEMENT

The authors declare that they have no competing interests.

## Supporting information


Table S1.

Table S2.

Table S3.

## Data Availability

Microarray datasets supporting the results of this study are available in the GEO database (https://www.ncbi.nlm.nih.gov/geo/).
